# Long-Term Assessment of Aesthetic Results in Omphalocele Repair with POSAS Scale

**DOI:** 10.1007/s00266-024-04101-2

**Published:** 2024-08-26

**Authors:** Eduje Thomas, Lorenzo De Benedetti, Giovanni Parente, Marco Di Mitri, Sara Maria Cravano, Simone D’Antonio, Tommaso Gargano, Mario Lima

**Affiliations:** IRCCS, Department of Paediatric Surgery of Bologna, S.Orsola Policlinic, Bologna, Italy

**Keywords:** Paediatric surgery, Omphalocele, Neonatal surgery, Congenital wall defects

## Abstract

**Introduction:**

Omphalocele (OM) is a congenital defect of the abdominal wall. The main goal of the surgical management is the survival of the neonate. However, the residual scar following the surgery can be extremely burdensome and negatively impact the quality of life (QoL) of these patients. The aim of this study is to assess the cosmetic results of the surgical treatment, the level of satisfaction of patients and surgeons, and the influence of the scar on the QoL of the patient.

**Materials and methods:**

We conducted an observational retrospective cross-sectional study collecting all data regarding patients born with OM, operated at our Centre between 1998 and 2021. The cosmetic results of the surgical repair were evaluated using the validated Patient and Observer Scar Assessment Scale (POSAS). The assessment of the quality of life determined by the presence of the scar was conducted using PedQL 4.0. At last, the patients were visited by two paediatric surgeons and a medical student, which then scored the cosmetic result of the scar. Statistical analysis was conducted with Spearman linear correlation and Mann–Whitney test. A *P*-value below 0.05 was considered statistically significant.

**Results:**

In our study, we included a total of 19 patients, with a mean of 12 years of age at the time of the evaluation. The parameters with the major influence on the patient’s general opinion of the scar were stiffness, thickness, and irregularity. We discovered significant differences in median values of all scores between the giant OM group and the nongiant OM group, in favour of the latter. Finally, we found a low grade of concordance between PedsQL filled by parents and patients.

**Conclusion:**

The POSAS scale is a valid, feasible, and reliable tool for the assessment of the aesthetic outcome of surgical procedures. The original size of the defect is the most important factor acting on the result. However, it is crucial that any decision on plastic surgery to improve the looks of the scar must be postponed to the adult age of the patient.

**Level of Evidence IV:**

This journal requires that authors assign a level of evidence to each article. For a full description of these Evidence-Based Medicine ratings, please refer to the Table of Contents or the online Instructions to Authors www.springer.com/00266.

## Introduction

**Fig. 1:**
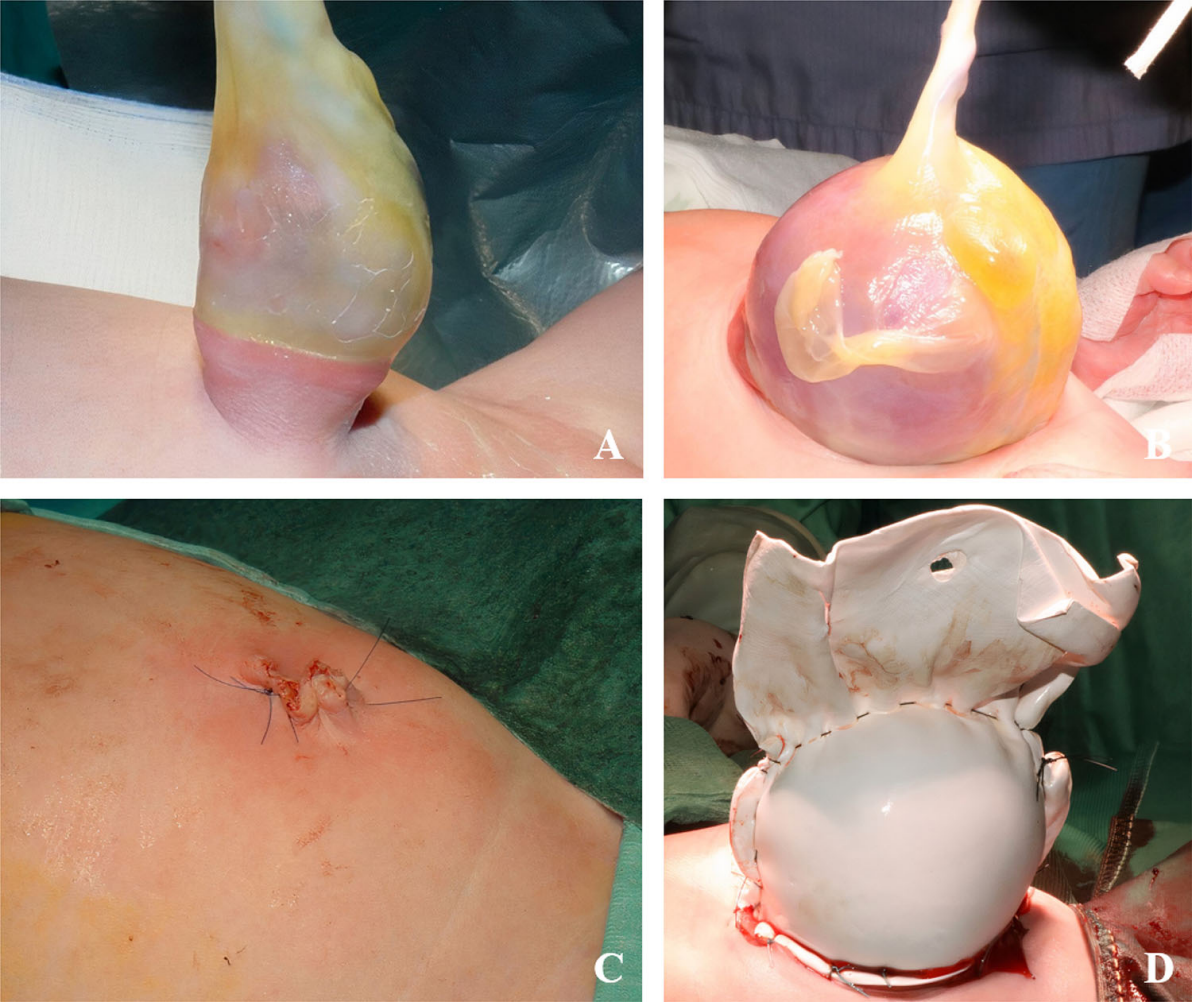
**A** Small omphalocele with herniation of viscera into a three-layered sac. **B** Giant OM with herniation of the liver. **C** Postoperative outcome of a small omphalocele subjected to a primary fascial closure. **D** Giant omphalocele dressed in a Gore-Tex silo according to Schuster’s technique for a staged closure

Omphalocele (OM) is a congenital defect of the abdominal wall, caused by a failure of the abdominal contents to return into their cavity by week 10 of gestation [1]. Differently from in gastroschisis, the defect is located on the median line and the viscera herniate into a three-layered sac, formed by visceral peritoneum, Wharton jelly and amnion [2, 3]. The size of the defect beneath the OM is variable, ranging from a few centimetres (cm) to over 8–9 cm, and represents the criteria for a classification that separates OMs into hernias of the cord, small, medium, and giant defects. Based on the size, the sac might contain viscera including bowels, liver, bladder, stomach, spleen, ovaries, and testis [4]. Usually diagnosed prenatally thanks to foetal ultrasound, an accurate assessment of associated anomalies is paramount as these represent the most important survival prognosticator. The anomalies most frequently encountered are chromosomic and genetic, followed by anomalies involving heart, central nervous system, urinary tract, genitalia, limbs, and digestive tract. In 12% of cases, OM can be found as a component of a syndrome, among which the most frequent is Beckwith–Wiedemann syndrome. In 30% of cases, the neonates might present also with a variable degree of pulmonary hypoplasia [5–8]. The main goal of the surgical management is to guarantee the survival of the neonate, as OM is a potentially life-threatening condition if not properly managed, mostly due to the infective risk. Surgical options can be divided into two main categories: immediate primary closure and delayed staged closure. The possibility of a primary closure depends on the size of the defect. Small defects are easier to close in the neonatal period with the use of a prosthesis, when necessary [9, 10]. Furthermore, the associated loss of abdominal domain, which can lead to the risk of compartmental syndrome when a tight closure is forced, is minimal in small defects. Large and giant OMs are always marked by a pronounced lack of abdominal domain and for such reason, pose giant hurdles to paediatric surgeons worldwide (Fig. 1).

In 1967, Schuster proposed to use a Silastic silo sutured to the abdominal wall after excision of the sac, to allow gradual reduction of the abdominal contents [11]. However, during the years, several nonoperative techniques have gained popularity, all bearing the same principle: the application of substances on the sac to induce the development of an eschar, which epithelizes over time leaving a ventral hernia that can be repaired later in life. The ventral hernia repair might be obtained with a primary fascial closure or may necessitate a mesh repair [11, 12].

**Fig. 2:**
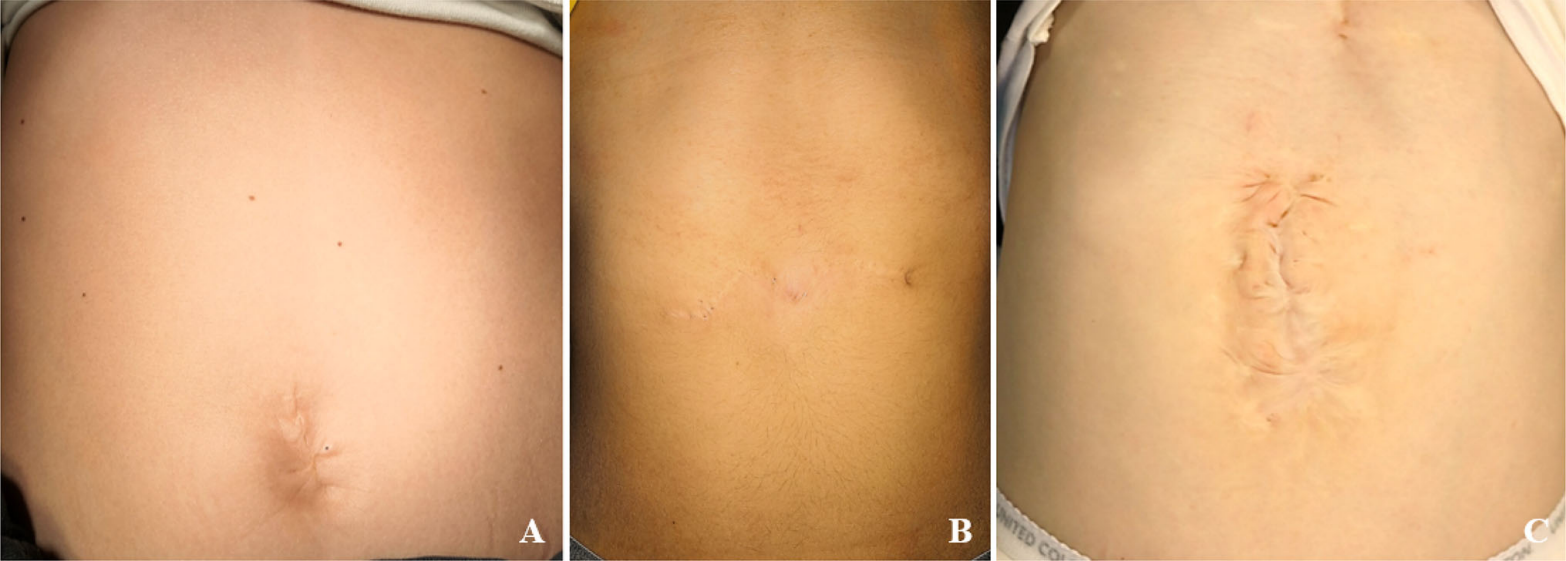
Postoperative cosmetic results after omphalocele correction. **A** Small omphalocele. **B** Larger defect. **C** Giant omphalocele

Independently from the adopted technique, the residual scar following the surgery can be extremely burdensome and negatively impact the quality of life (QoL) of these patients (Fig. 2). The aim of this study is to assess the cosmetic results of the surgical treatment, the level of satisfaction of patients and surgeons, and the influence of the scar on the QoL of the patient.

## Materials and Methods

### Study Population

Our study is an observational cross-sectional study in which we gathered all data regarding patients born with OM, regardless of the size of the defect, operated at a tertiary centre of Paediatric Surgery between 1998 and 2021. Demographic, clinical, and surgical data were collected consulting surgical and clinical registers, and all patients were contacted by phone to schedule a visit in our outpatient clinic. Therefore, the inclusion of the patients in the study was bound to the availability to attend our clinic for an evaluation.

During the visit, we obtained the informed consent to the participation in the study and then administered a first questionnaire for the scar assessment to parents/legal guardians and patients, when age permitted. Then, we administered a second questionnaire for the evaluation of the QoL to both parents/legal guardians and patients, with the aim of identifying the life quality related to the presence of the scar. At last, the patients were visited by two clinicians, a paediatric surgeon, and a medical student, which scored the cosmetic result of the scar.

### Scar Assessment

At the current state of the art, two main scar evaluation tools have been validated in the literature. First described in 1990 by Sullivan et al., the Burn Scar Index, often referred to as the Vancouver Scar Scale (VSS), has been the most reliable, objective, and universal method for assessing scars for a long time [14]. Initially conceived for the evaluation of burn scars, the VSS examines four components of the scar: pigmentation, vascularity, pliability, and height. Each parameter is rated from 0 to 3, except pliability which is rated from 0 to 5, with the severity increasing with the score. Much of the approval received by the VSS is also due to its statistically proved interrater reliability, which even improved as the clinicians gained major familiarity with the scale. However, the same authors recognized the lack of an appraisal of symptoms, such as pain and itching that are important in the treatment of these scars.

**Fig. 3:**
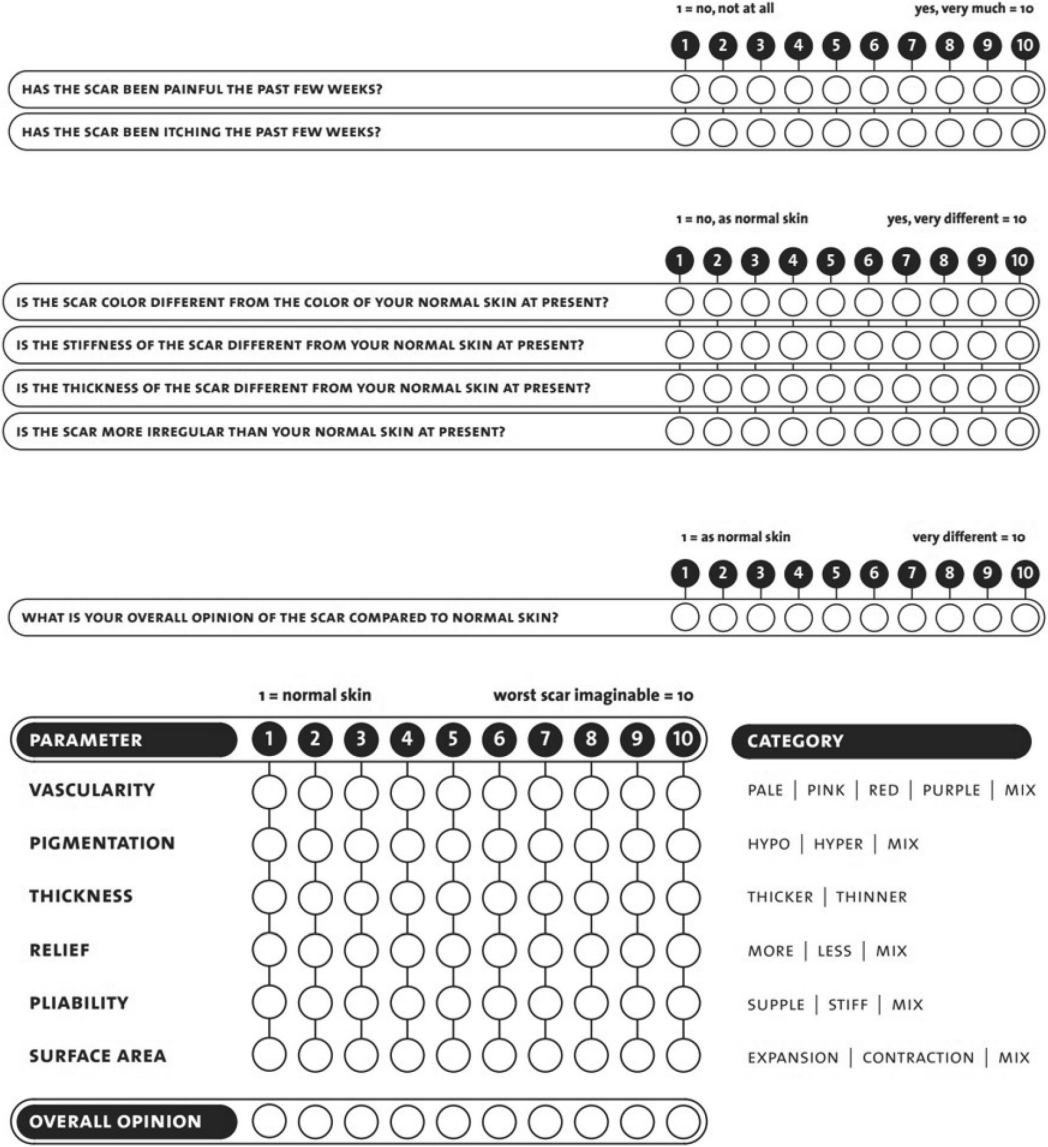
The Patient and Observer Scar Assessment Scale 2.0 (POSAS)

To overcome the limitations of the VSS, in 2004 Draaijers et al. proposed the Patient and Observer Scar Assessment Scale (POSAS) for a subjective and objective evaluation of all types of scars (Fig. 3) [15]. It entails two numeric scales: the Patient Scar Assessment Scale (PSAS), which must be filled by the patient, and the Observer Scar Assessment Scale (OSAS), reserved for the evaluating clinician. The PSAS contains six items, and compared to the VSS, it also rates symptoms such as pain and itch. The OSAS gauges vascularization, pigmentation, thickness, relief, and pliability. The evaluation of every parameter must be done in comparison with an area of normal skin. Each item of both scores is numerically rated from 1 to 10, and in addition to the scar assessment, both patients and observers are asked to give a general opinion on the appearance of the scar from 1 to 10, in which a score of 10 corresponds to the worst possible cosmetic result.

### Quality of Life Assessment

The evaluation of the impact of the scar on the QoL was done with the Paediatric Quality of Life Inventory 4.0 (PedsQL). PedsQL is a validated and reliable tool for evidence-based research, shaped on the age of the patient, that appraises the physical and psychosocial functioning of the patient with a questionnaire containing 21 or 23 questions. Both patients and parents/legal guardian receive a custom-made questionnaire investigating four macro areas regarding the life of the patient: physical, emotional, social, and school functioning. A score from 0 to 4 is assigned to each question, with a score of 4 corresponding to the worst possible outcome. Each score is then converted with an inverted scale, according to which 0=100, 1=75, 2=50, 3=25, 4=0, and the final score for each category of questions is obtained by calculating the average. In previous studies, a score of 83 has been identified as the lower limit for a normal QoL in healthy patients, whereas a score of 77 represents the lower limit in individual with chronic conditions [16].

In order to avoid any influence, patients and parents/legal guardians were asked to fill the PedsQL questionnaire separately, as well as Observer 1 and 2 conducted their assessments and filled the OSAS in separate instances. To conclude the evaluation, patients, parents/legal guardians, and both observers were asked to note if they would have recommended plastic surgery to improve the cosmetic result of the scar.

### Statistical Analysis

Demographic data, gestational age and weight at birth, and PedsQL scores were expressed with mean values and standard deviations. Spearman linear correlation was used to identify which parameters mostly influenced the general opinion of patients and observers in the POSAS. The same analysis was conducted to verify the interrater accordance of the OSAS among the two observers and of PedsQL between patients and parents/legal guardians. At last, Mann–Whitney test was used to compare the results of the POSAS scores in patients with a normal or impaired QoL, measured with PedsQL, and in patients with or without a giant OM, and the results of PedsQL in patients with or without a giant OM. For the purpose of the study, in the absence of a universally accepted definition of giant OM, we decided to include in this group defects larger than 5 cm or with a herniation of the liver [13]. A *P*-value below 0.05 was considered statistically significant.

The paper was written in accordance with the STrengthening the Reporting of OBservational studies in Epidemiology (STROBE) checklist.

## Results

In our case series, thirty-four patients were operated at birth for OM between 1998 and 2001 and hence considered eligible for the study. Three patients died in the early postoperative period during the hospital stay, because of important life-threatening comorbidities. We contacted the remaining thirty-one patients, of which three deceased in the first years of life, four were unreachable, and five refused to participate in the study.

Our final cohort was made by 19 patients, 8 (42.1%) females and 11 (57.9%) males, with a mean age of 12 (1–24 years). Mean gestational age at birth was 37.0 ± 1.9 weeks and mean weight at birth was 3.0 + 0.6 kg. Reported comorbidities were Beckwith–Wiedemann syndrome in 3 patients (15.8%), congenital heart diseases in 5 patients (26.3%), and Meckel diverticulum in 2 patients (10.5%). In compliance with the definition chosen by our research group, 6 patients (31.6%) presented with a giant OM, whereas 13 patients (68.4%) had smaller defects. In 3 patients with giant OM, an immediate primary repair was obtained thanks to adequate stretching of the abdominal wall prior to the myofascial closure. In the remaining 3 patients a Gore-Tex silo was mounted, to allow gradual reduction of the abdominal contents, with the final closure obtained in 15–21 days. Only one of these patients was later subjected to umbilicoplasty, 7 years after the definitive closure. One of the patients underwent another laparotomy in the first years of life because of intestinal volvulus.

Mean value scores and standard deviations for each item of both PSAS and OSAS of both observers are shown in Table 1.

**Table 1:** Mean value scores and standard deviations for each item of both PSAS and OSAS of both observers

Parameters	PSAS	OSAS 1	OSAS 2
Pain	1 + 1	–	–
Itching	1 + 1	–	–
Vascularity	–	3 + 2	3 + 1
Pigmentation	3 + 2	3 + 2	3 + 1
Stiffness/Pliability	5 + 3	5 + 3	4 + 3
Thickness	5 + 3	4 + 3	4 + 2
Irregularity/Relief	5 + 4	4 + 3	4 + 3
Surface Area	–	4 + 3	4 + 3
Total	19 + 10	23 + 13	23 + 12
Overall	5 + 3	4 + 3	5 + 3

Total scores of PSAS and OSAS are shown in relation to the mean total scores of PedsQL for each patient in Table 2. It is important to remember that total scores of the POSAS and of PedsQL are related to the outcomes in an opposite manner, as the maximum score of the POSAS corresponds to the worst possible outcome, whereas higher scores of PedsQL indicate a better QoL.

**Table 2:** Total scores of PSAS, OSAS, and PedsQL for each patient

Patient	PSAS	OSAS 1	OSAS 2	PedsQL Patient	PedQL Parent
1	38	47	44	32	30
2	21	11	14	100	99
3	19	18	18	99	86
4	8	16	16	88	69
5	14	41	41	94	73
6	30	21	20	–	71
7	14	19	19	94	93
8	18	11	11	63	100
9	30	37	35	87	59
10	16	17	16	97	89
11	31	26	25	78	56
12	13	11	15	77	76
13	35	32	31	–	67
14	19	10	11	63	59
15	6	6	7	100	85
16	14	25	25	100	100
17	6	7	8	99	85
18	32	47	47	91	71
19	6	29	30	90	90
Mean + SD	19 + 10	23 + 13	23 + 12	85 + 18	77 + 18

When analysing which parameter mostly influenced the general opinion of the patient on the residual scar, we found a close relation between the global score and the stiffness (R^2^ = 0.64), the thickness (R^2^ = 0.67) and the irregularity (R^2^ = 0.86) of the scar. On the other hand, the remaining parameters showed a weaker correlation with the general opinion. We also found a high level of interrater concordance with a R^2^ correlation factor of 0.99, between observations made by both clinicians.

Comparing median values of the total scores in patients born with a giant OM and patients with smaller defects, we found statistically significant differences in the results of PSAS, OSAS 1, and OSAS 2. Significant differences were highlighted also in the comparison of PedsQL filled by parents in both groups (Table 3).

**Table 3:** Mean values of the total scores in patients born with and without a giant OM

Scale	Giant OM	Non-Giant OM	*P*-value
PSAS total	32 (19–38)	14 (6–30)	0.002
OSAS 1 total	35 (18–47)	16 (6–41)	0.010
OSAS 2 total	33 (18–47)	16 (7–41)	0.011
PedsQL	62 + 19	84 + 13	0.016

**Fig. 4:**
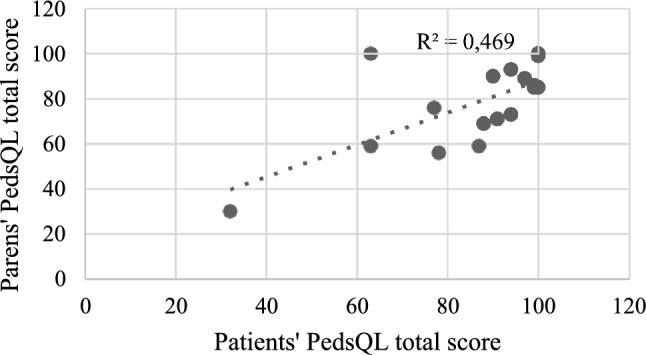
Correlation between PedsQL patients and parents

The results of PedsQL filled by parents and patients showed a low grade of correlation (R^2^ = 0.47) (Fig. 4). Dividing our cohort in two groups based on the QoL, using a global score above 83 of PedsQL as a cut-off to define a normal QoL, we found 12 patients (70.6%) with a normal QoL and 5 patients (29.4%) with an impaired QoL. Such results were not matched by the PedsQL filled by the parents/legal guardians, according to which only 9 patients (47.4%) had a normal QoL, while 10 patients (52.6%) presented with an abnormal QoL.

Statistical analysis showed a significant difference when comparing PSAS total scores in the two groups based on the PedsQL administered to parents/legal guardians (p = 0.033). However, no statistically significant difference was found when making the same comparison in the two groups based on the results of PedsQL filled by the patients, with a p value of 0.168.

## Discussion

Among the congenital defects of the abdominal wall, OM carries the highest difficulties regarding the surgical management. Over the decades, several techniques have been developed to achieve the survival of the patient, which remains the main goal in the treatment of OM. However, this condition not only represents a challenge in terms of treatment but also of long-term follow-up, due to important comorbidities, consequences of surgery, physical growth retardation and cosmetic outcomes. While the best approach is still debated, especially in OMs with an important defect, to our knowledge, no study has considered the opinion of the patients and parents about the aesthetic results.

Hijkoop et al. reported a delayed motor development in children with giant omphalocele and advocate the necessity of close monitoring of these children and early referral to physical therapy. The same authors evaluated parent-reported motor function, cognition, health status, QoL, and behaviour in school-aged children treated for OM. In a study cohort of 31 patients, thirteen per cent of parents were found to have a heightened perception of the child’s vulnerability, and this fear was mostly related to the exceptionality of the situation. Therefore, they concluded highlighting the importance of parental counselling and support as an aid to lower the level of perceived vulnerability.

As a matter of fact, the World Health Organization defines QoL as an individual’s perception of their position in life in the context of the culture and value systems in which they live and in relation to their goals, expectations, standards, and concerns (WHOQL Group 1995).

This holistic idea of QoL also embraces the concept that self-esteem is also based on the personal idea of our physical self and appearance, so a scar can be seen as an element of disruption. Moreover, the inability to satisfy the idea of a perfect body conveyed by media generates a social pressure that can lead to anxiety and negatively impact QoL. It is well known that cutaneous scarring is associated with social avoidance, anxiety, and depression [3, 4]

Besides creating a certain degree of psychologic discomfort, scars can impede physical functioning as it might generate a certain amount of inflexible tissue, which can compromise general functionality. Symptoms such as pain and pruritus come into play mostly when scars develop hypertrophic aspects or evolve into keloids and are often perceived as burdensome by patients. Because of the lack of an assessment of these features in the VSS, a renowned limitation also recognized by its original authors, we decided to rely on the POSAS for the evaluation of the scars. This score yields the advantage of a dual opinion, giving the floor to both patients and clinicians. However, the results emerging from our study seem to disprove the necessity of an evaluation of symptoms. The general opinion given by the patients about the scars was more closely related to parameters such as stiffness, irregularity, and thickness rather than the actual presence of symptoms.

Moreover, the POSAS showed high grade of interrater concordance, one of the acclaimed qualities of the VSS. Although being conducted by observers with a completely different experience, a medical student and a paediatric surgeon, the evaluation of the clinicians produced same results in almost the totality of cases, making it a reliable and feasible tool also in unexperienced hands.

As expected, the evaluation of the cosmetic results in patient with a giant OM is remarkably different from those with smaller defects, tending towards a more negative judgement in the assessment conducted by patients and both observers. Giant OMs pose the major hurdles when it comes to achieving an acceptable final aesthetic result. Since Ahlfeld first described in 1899 the application of a substance on the sac to promote cicatrisation, the “paint and wait” approaches have progressively gained popularity being currently adopted in many leading clinical centres. The delayed surgical procedure to repair the fascial defect and close the primary ventral hernia might require advanced techniques, and it is not uncommon to request the participation of a plastic surgeon to guarantee the best possible result.

Regarding the general QoL, it does not strike as surprising that patients who presented with a giant OM report a worse QoL compared to those with smaller defect, and such data remain persistent also in the parents’ opinions. Future QoL in OM patients can be impaired by abdominal problems, nutritional deficiencies, growth retardation, gastroesophageal reflux disease, and neurodevelopmental issues. Several studies report up to 30 % of incidence of chronic abdominal pain without patients receiving a diagnosis of small bowel adhesions or being reoperated [2, 19, 20]. Anthropometric features in these patients are persistently lower than their peers throughout the first two years of age, whereas mental development is comparable with reference norms [21]. However, C. van Eijck et al. reported no significant differences in gastrointestinal disorders between patients with giant OM and minor OM and also a good general QoL in both groups, comparable to healthy young adults [22].

It must be stated that, in our study, the focus was placed on how QoL was impaired by the scar with little regards to other sequelae. The comparative analysis of the PSAS score in patient with normal or altered QoL, defined as a total score below 83, revealed significantly lower scores in the latter. Such result proves the efficacy of both questionnaires in pinpointing those in which scars cause the highest degree of impairment.

Furthermore, it is well known that parents often have an enhanced perception of bothers and difficulties concerning the life of their little ones. Such tendency is also reflected by the results obtained from the PedsQL questionnaires, in which we found a low level of correlation between total score reported by patients and parents. As a matter of fact, our results prove that parents and patients agree on the influence of the scar on the QoL in less than 50% of cases. The parley on surgery for cosmetic purposes in children is under the spotlight nowadays. Singh et al. denounced how teenagers are getting access to cosmetic surgery in larger numbers compared to the past and call for the importance of the presence of a parent during consultation and informed consent [23]. Del Rio et al. analysed which factors may be involved in the decision of cosmetic surgery for children and adolescents with the assumption that only procedures that serve the “objective interest” in terms of a sound mental health in the teenage years can be deemed ethically acceptable. They highlighted the factors identified, such as age, maturity, psychological and emotional conditions, motivations of the minors, and the opportunity to procrastinate the operation. They concluded that physicians and parents are compelled to act in the minor’s best interest [24].

This fully brings us into the debate over transitional care, stressing how fundamental it might be to wait for the patients to reach for the age of maturity before taking any decision on whether to undergo plastic surgery to improve the residual scar. Otherwise, based on parents’ decision, patients could be subjected to another surgical procedure, and they might regret later in adulthood.

### Limitations of the Study

We are fully conscious of a series of limitations that hamper the strength of our findings and call for a cautious interpretation. At first, it would be sensible to perform such a study on a larger sample size to increase the statistical significance of any conclusion. Nevertheless, we think our data could be an interesting and encouraging preliminary result for further investigations such as multicentric studies. Furthermore, the point of view of the paediatric patients might suffer from bias related to their age. It might be interesting to have a new point of view of these patients when they reach adulthood. At last, the cross-sectional and retrospective nature of the study represent a limit as patients were assessed at different ages, while it would be preferable to follow a cohort of patients and evaluate the participants at the same age in adulthood.

## Conclusion

As the major efforts in the follow-up of patients treated for OM are gradually shifting towards the long-term distance, several studies have been conducted to identify areas of greater interest, where our focus must be placed. This study sheds light on one of the major concerns of patients and parents, the cosmetic result. The POSAS scale is a valid, feasible, and reliable tool for the assessment of the aesthetic outcome of surgical procedures. The original size of the defect is the most important factor acting on the final result. However, it is crucial to weigh up any decision on plastic surgery to improve the looks of the scar, in order to act in the best interests of the minor.
